# Clinical and Radiographic Outcomes of Two-Stage Allograft-Augmented Fixation Versus Conventional Posterior Segmental Stabilization for Screw Loosening

**DOI:** 10.3390/jcm15145408

**Published:** 2026-07-10

**Authors:** Can Sezer, Mehmet Volkan Harput, Ridvan Acikalin, Eray Polat, Mahmut Ferat, Ismail Istemen, Durdu Mehmet Babaoglan, Emre Bilgin, Aykut Sezer

**Affiliations:** 1 Department of Neurosurgery, Adana School of Medicine, University of Health Sciences, Adana 01230, Turkey; 2 Department of Neurosurgery, Adana City Training and Research Hospital, Adana 01000, Turkey; 3 Department of Neurosurgery, İstinye University, İstanbul 34010, Turkey; 4 Department of Neurosurgery, Dr. Ersin Arslan Training and Research Hospital, Gaziantep 27010, Turkey

**Keywords:** two-stage fixation, allograft augmentation, posterior segmental stabilization, screw loosening, sagittal balance

## Abstract

**Background:** Successful spinal stabilization depends not only on early mechanical fixation but also on durable biological integration at the bone–implant interface. This study aimed to compare a two-stage allograft-augmented fixation strategy, intended to promote biological fixation, with conventional posterior segmental stabilization (PSS) in the treatment of screw loosening following multilevel spinal surgery for degenerative spine pathology. **Methods:** This retrospective comparative study included 40 patients who underwent multilevel spinal stabilization between January 2022 and February 2025, with follow-up completed by November 2025. Patients were divided into a conventional PSS group (*n* = 28) and a two-stage allograft-augmented fixation group (OI-focused strategy, *n* = 12). Demographic characteristics, comorbidity burden, length of hospital stay, complications, Visual Analog Scale (VAS), the Oswestry Disability Index (ODI), and sagittal radiographic parameters—including lumbar lordosis (LL), sagittal vertical axis (SVA), sacral slope (SS), and pelvic incidence (PI)—were evaluated preoperatively and at 1, 6, and 9 months postoperatively. **Results:** The groups were similar in terms of age, sex, body mass index, smoking status, and number of operated levels. Despite the significantly higher Charlson Comorbidity Index and Elixhauser Comorbidity Index scores in the OI-focused group, hospital stay was significantly shorter. VAS and ODI scores were comparable preoperatively and at 1 month; however, significantly better pain and functional outcomes were observed in the OI-focused group at 6 and 9 months. Radiologically, SVA was significantly lower in the OI-focused group at 6 and 9 months. Late differences were also observed in LL and SS, while PI remained similar between groups. Fusion-related findings were interpreted cautiously because fusion was assessed using plain radiographs and osseointegration was not directly measured. **Conclusions:** The two-stage allograft-augmented fixation strategy was associated with better mid-term pain control, improved functional recovery, and better preservation of selected sagittal alignment parameters compared with conventional PSS. However, these findings should be interpreted cautiously due to the retrospective design, limited sample size, imbalanced groups, absence of direct osseointegration measurement, and lack of CT-based fusion analysis.

## 1. Introduction

Successful outcomes in spine surgery depend on both early mechanical stability and sustained biological fixation at the implant–bone interface. In degenerative spinal disorders requiring instrumented stabilization, early postoperative recovery is largely attributed to the mechanical performance of the construct; however, mid- and long-term success is also associated with biological integration, maintenance of fixation, preservation of alignment, and progression to fusion [1,2,3,4].

Osseointegration (OI) is classically defined as a direct structural and functional connection between living bone and the surface of a load-bearing implant without an intervening fibrous layer [1]. Although the concept was initially emphasized in dental and orthopedic implantology, it has gained increasing importance in spine surgery with the development of titanium-based materials, porous surface architectures, 3D-printed cages, and bioactive surface modifications designed to enhance bone ingrowth and biological fixation [3,4,5,6,7,8].

Conventional posterior segmental stabilization (PSS) remains one of the most commonly used surgical strategies in degenerative spine disease, as it provides strong and effective early mechanical support [2]. However, in multilevel fusion procedures and in patients with poor bone quality or high comorbidity burden, complications such as pedicle screw loosening, micromotion, implant failure, and pseudoarthrosis may adversely affect clinical outcomes and radiographic durability [9,10].

Meanwhile, the importance of global and regional sagittal alignment in postoperative outcomes has become increasingly evident. Parameters such as sagittal vertical axis, lumbar lordosis, sacral slope, and pelvic incidence are closely associated with pain, disability, energy expenditure, and health-related quality of life [11,12,13,14]. Therefore, both clinical outcomes and alignment-related radiographic parameters should be considered when evaluating biologically advantageous instrumentation strategies.

Despite the rapidly expanding literature on biomaterials and cage technologies, clinical data comparing biologically oriented fixation strategies with conventional PSS remain limited. In patients undergoing multilevel surgery for degenerative spine pathology, treatment of screw loosening has traditionally involved revision with conventional posterior segmental stabilization. The aim of this study was therefore to compare conventional single-stage PSS with a two-stage allograft-augmented fixation strategy designed to support biological fixation at the bone–implant interface. Because osseointegration was not directly verified histologically, biomechanically, or by quantitative bone–implant interface imaging, the term “OI-focused strategy” is used in this manuscript as a descriptive term for the surgical strategy rather than as proof of a directly measured biological phenomenon.

## 2. Materials and Methods

### 2.1. Study Design

Patients who underwent posterior long-segment fusion between January 2022 and February 2025, with follow-up completed by November 2025, were retrospectively analyzed. This study was designed as a retrospective comparative clinical analysis of patients who developed screw loosening after multilevel spinal stabilization due to degenerative spinal pathology. Poor bone quality or osteoporosis was suspected clinically and radiographically in this cohort; however, complete bone mineral density (BMD)/DXA data were not uniformly available and were therefore not used as an inclusion criterion. Following institutional ethics committee approval, patients older than 18 years with at least 9 months of clinical and radiographic follow-up were included. Patients with a history of tumor, infection, trauma, prior pelvic surgery, or incomplete clinical/radiographic data were excluded.

The selection of the surgical strategy was based on clinical, radiographic, and intraoperative criteria, including radiographic evidence of pedicle screw loosening, persistent pain and disability after previous multilevel stabilization, suspected poor bone quality on routine imaging, the number of involved levels, the need for revision stabilization, and intraoperative assessment of screw purchase. Patients with severe loosening, compromised screw purchase, and a need for biological augmentation at the bone–implant interface were considered for the two-stage allograft-augmented strategy. The final surgical decision was made by the treating surgical team according to patient-specific factors; because treatment allocation was not randomized, selection bias cannot be excluded.

A total of 40 patients were included. Twenty-eight patients underwent revision with conventional posterior segmental stabilization (PSS group), while twelve patients were treated with a two-stage allograft-augmented fixation strategy intended to promote biological fixation (OI-focused group).

### 2.2. Clinical Evaluation

The following variables were assessed: age, sex, body mass index, smoking status, number of instrumented levels, Charlson Comorbidity Index (CCI), Elixhauser Comorbidity Index (ECI), length of hospital stay, complications, and in-hospital mortality.

Pain severity was assessed using the Visual Analog Scale (VAS), and functional disability was evaluated using the Oswestry Disability Index (ODI). Clinical and radiological outcomes were recorded preoperatively and at 1, 6, and 9 months postoperatively.

### 2.3. Surgical Technique

In the PSS group, standard larger-diameter titanium pedicle screw-rod constructs were used in a single-stage posterior segmental stabilization procedure. In the OI-focused group, standard clinically available larger-diameter titanium pedicle screws were placed with circumferentially impacted cancellous allograft bone around the screw trajectory and screw–bone interface. No manufacturer-specific porous or 3D-printed pedicle screw technology, dedicated osseointegrative surface coating, bioactive screw coating, cement augmentation, or other biomaterial surface modification was used. A two-stage protocol was applied in this group: screws and allograft augmentation were placed initially without rod connection, and rod placement was performed six months later. Therefore, the term “OI-focused” refers to the biological rationale of allograft augmentation and staged load sharing at the bone–implant interface rather than to a directly measured or proprietary osseointegration technology.

**Figure 1:**
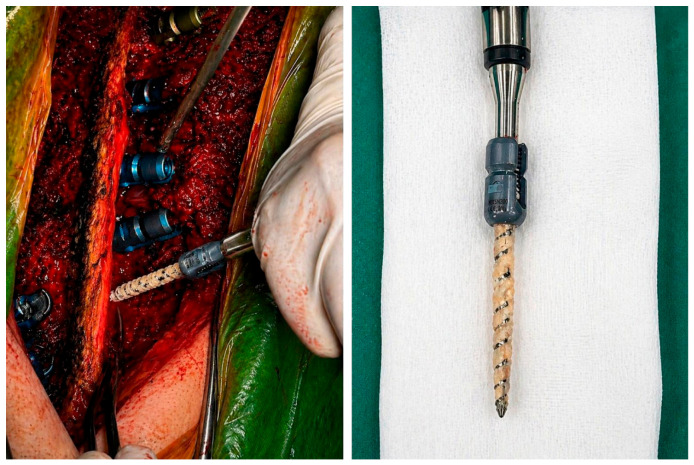
Intraoperative view of an allograft-augmented pedicle screw used as part of the two-stage OI-focused fixation strategy.

**Figure 2:**
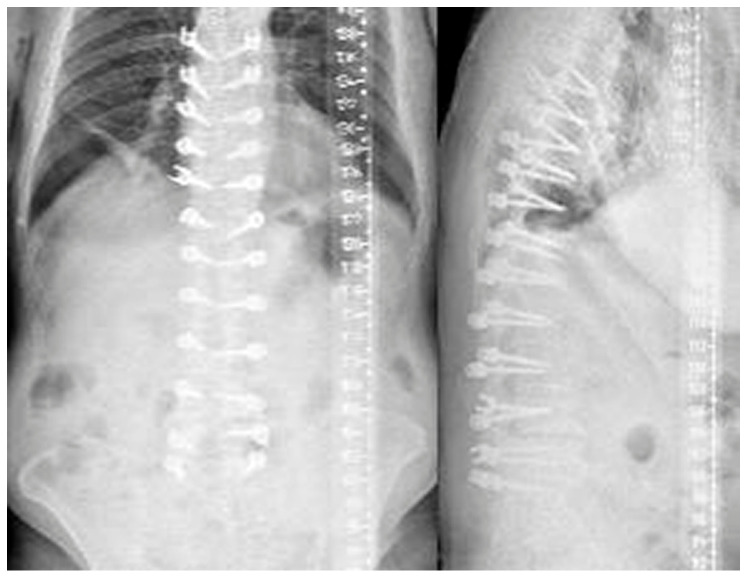
Pre- and postoperative AP and lateral radiographs showing multilevel posterior stabilization and alignment restoration.

In both groups, the primary surgical goals were adequate decompression and stabilization; however, the two-stage allograft-augmented strategy specifically aimed to create a more favorable biological environment at the bone–implant interface (Figure 1 and Figure 2).

### 2.4. Radiological Evaluation

Spinopelvic parameters measured included sagittal vertical axis (SVA), lumbar lordosis (LL), sacral slope (SS), and pelvic incidence (PI).

**Figure 3:**
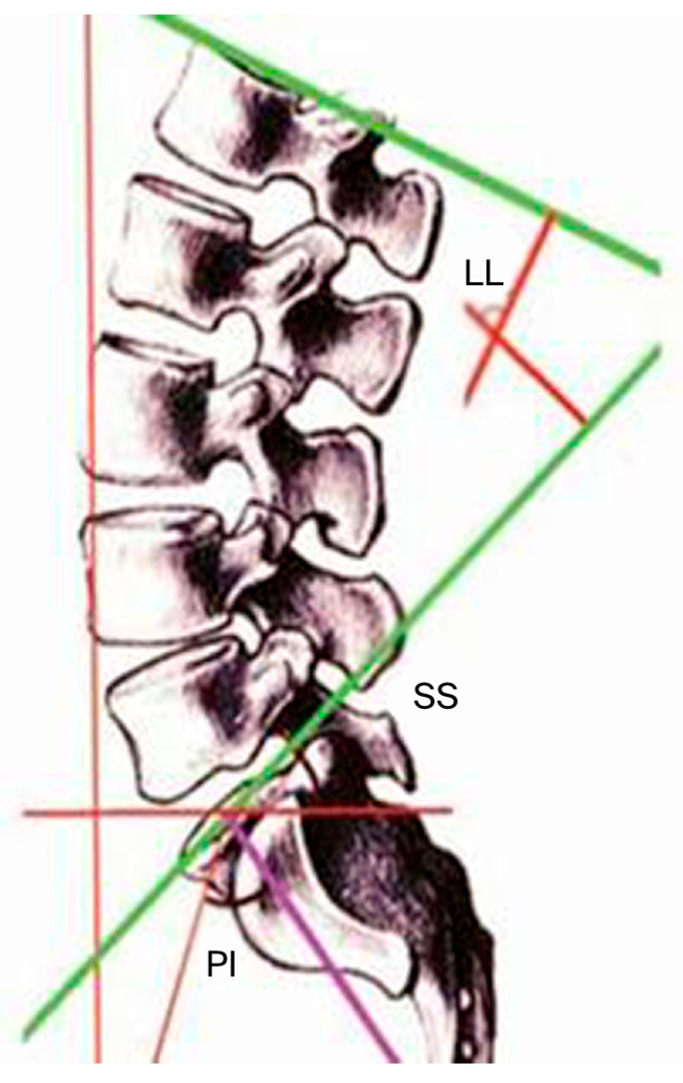
Schematic illustration of spinopelvic parameters.

**Figure 4:**
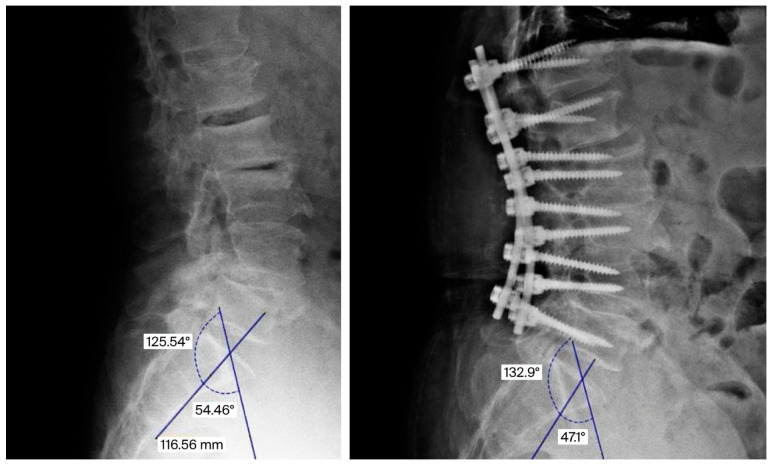
Lateral radiographs demonstrating measurement of lumbar lordosis.

- SVA: horizontal distance between the C7 plumb line and the posterosuperior corner of the sacrum;
- LL: measured between the superior endplates of L1 and S1;
- SS: angle between the sacral endplate and the horizontal plane;
- PI: angle between a perpendicular line from the midpoint of the sacral endplate and a line connecting this point to the femoral head axis (Figure 3 and Figure 4).

Measurements were recorded preoperatively and at 1, 6, and 9 months postoperatively.

Fusion status on plain radiographs was evaluated using the Bridwell fusion grading system:

- Grade I: Complete fusion;
- Grade II: Graft remodeling present, no radiolucent lines;
- Grade III: Radiolucency present;
- Grade IV: No fusion.

All radiographic measurements were performed independently by two observers who were not involved in the surgical decision-making process. Because implant configuration was visible on radiographs, complete blinding to treatment group was not feasible; however, observers were blinded to clinical outcome scores during measurement. Disagreements were resolved by repeat measurement and consensus review. Formal inter-observer reliability statistics, including intraclass correlation coefficients (ICC), were not calculated because separate observer-level datasets were not uniformly preserved for all radiographic parameters in this retrospective cohort; this is acknowledged as a methodological limitation.

### 2.5. Statistical Analysis

Continuous variables were primarily expressed as mean values, and categorical variables were expressed as numbers and percentages. Because this retrospective study was based on an anonymized aggregated dataset, standard deviations and effect-estimate confidence intervals could not be uniformly reconstructed for all variables; therefore, this limitation is acknowledged as a reporting limitation. *p*-values were reported for statistical comparisons; confidence intervals were not calculated for *p*-values because *p*-values themselves do not have confidence intervals. Where appropriate and calculable, 95% confidence intervals were provided for the corresponding effect estimates as measures of precision. Comparisons were performed using Student’s *t*-test or Mann–Whitney U test as appropriate. Categorical variables were analyzed using chi-square or Fisher’s exact test. Statistical analyses were performed using IBM SPSS Statistics for Windows, version 29.0 (IBM Corp., Armonk, NY, USA). A *p*-value < 0.05 was considered statistically significant. Missing data were minimal across the primary clinical and radiographic variables due to strict inclusion criteria requiring complete follow-up; therefore, no imputation methods were applied, and analyses were performed on complete-case data. No formal prospective a priori power calculation was performed because this was a retrospective fixed-cohort study. A G*Power (Version 3.1.9.7)-compatible sensitivity analysis for the available group sizes (PSS, *n* = 28; OI-focused, *n* = 12; alpha = 0.05; power = 0.80) indicated that the study was powered only to detect large between-group effects (approximately Cohen’s d = 0.99). Because multiple exploratory comparisons were performed across clinical and radiographic endpoints, no formal multiplicity adjustment was applied, and no claim of confirmatory statistical significance is made. Multivariable regression was not performed because the small and imbalanced sample size, particularly for the OI-focused group (*n* = 12), would make model estimates unstable and prone to overfitting. Accordingly, all *p*-values and confidence intervals should be interpreted as exploratory, and the findings should be considered hypothesis-generating rather than definitive.

## 3. Results

### 3.1. Baseline and Perioperative Findings

The mean age was 60.8 years in the PSS group and 62.3 years in the OI-focused group, with no significant difference between the groups. Gender distribution, body mass index, smoking status, and number of surgical levels were also comparable. Despite similar baseline demographic characteristics, the comorbidity burden was significantly higher in the OI-focused group. The mean Charlson Comorbidity Index (CCI) was 0.79 in the OI-focused group and 0.68 in the PSS group (*p* = 0.043). The mean Elixhauser Comorbidity Index (ECI) values were 1.81 and 1.32, respectively (*p* = 0.028). Nevertheless, the length of hospital stay was significantly shorter in the OI-focused group (3.1 vs. 5.7 days; *p* = 0.035). Complication rates were 8.3% in the OI-focused group and 14.3% in the PSS group, without statistical significance. No in-hospital mortality was observed in either group (Table 1).

### 3.2. Clinical and Functional Outcomes

Preoperative VAS scores were similar between groups (PSS: 8.12; OI: 8.22; *p* = 0.564). At 1 month postoperatively, both groups demonstrated marked improvement, with no significant difference (3.45 vs. 3.91; *p* = 0.132). At 6 months, VAS scores were significantly lower in the OI-focused group compared to the PSS group (1.47 vs. 2.21; *p* = 0.026), and this difference persisted at 9 months (1.46 vs. 2.19; *p* = 0.023).

A similar trend was observed for ODI scores. Preoperative ODI values were 54.48 in the PSS group and 56.72 in the OI-focused group (*p* = 0.316). At 1 month, the groups remained comparable (26.53 vs. 28.32; *p* = 0.361). At 6 months, ODI scores were significantly lower in the OI-focused group (17.12 vs. 21.68; *p* = 0.038), and this difference was maintained at 9 months (17.11 vs. 21.54; *p* = 0.043) (Table 2).

### 3.3. Radiographic Outcomes

Preoperative LL values were similar between groups (PSS: 68.9°; OI: 69.2°; *p* = 0.817). No significant differences were observed at 1 and 6 months, although the comparison at 6 months approached significance (*p* = 0.057). At 9 months, LL differed significantly between groups (67.7° vs. 61.7°; *p* = 0.024).

SVA values were similar preoperatively and at 1 month. At 6 months, SVA was significantly lower in the OI-focused group (22.7 mm vs. 25.6 mm; *p* = 0.043), and this trend persisted at 9 months (22.8 mm vs. 25.9 mm; *p* = 0.038).

SS values were comparable preoperatively and at 1 month, approached significance at 6 months (*p* = 0.053), and showed a significant difference at 9 months (PSS: 51.7°; OI: 48.2°; *p* = 0.042). Pelvic incidence did not differ significantly between groups at any time point (Table 3).

Fusion-related outcomes were reassessed and are now presented in tabular form rather than as a 3D bar chart. The standard clinical and radiographic follow-up schedule was 1, 6, and 9 months, and Bridwell fusion grades were available at all three follow-up time points. Because fusion was evaluated using plain radiographs rather than CT, these data should be interpreted cautiously. Available Bridwell grades at 1, 6, and 9 months are shown in Table 4. Exploratory Mann–Whitney U testing showed no statistically significant between-group difference at 1 month (*p* = 0.205), whereas statistically significant between-group differences were observed at 6 months (*p* = 0.026) and 9 months (*p* = 0.043); however, these findings should not be interpreted as definitive evidence of superior fusion because CT-based confirmation was not available (Table 4).

## 4. Discussion

This study suggests that a two-stage allograft-augmented fixation strategy may be associated with clinically meaningful advantages over conventional PSS in multilevel degenerative spine surgery, particularly beyond the early postoperative period. Although both groups exhibited substantial early improvement, patients treated with the OI-focused strategy demonstrated significantly lower VAS and ODI scores at 6 and 9 months. This temporal pattern is consistent with the intended biological rationale of reducing micromotion and improving the bone–implant environment over time; however, the present study cannot directly prove osseointegration or separate its contribution from the effects of staged fixation, altered load-sharing mechanics, rehabilitation protocols, or other unmeasured confounders.

Recent literature supports the idea that implant surface properties, graft augmentation, and material architecture can influence the biological behavior of spinal constructs. Titanium implants combined with bone graft have been associated with osteoconductive potential, bone ingrowth, and favorable mechanical compatibility in selected spinal applications [5,6,7,8,15,16]. Accordingly, the mid-term clinical outcomes observed in this series may reflect the cumulative effect of allograft augmentation, staged instrumentation, and a more favorable bone–implant environment. Nevertheless, these mechanisms remain inferential in the present study because no histological, pull-out, or quantitative interface-imaging assessment was performed.

A notable finding in the present cohort is that the OI-focused group, despite having a significantly higher comorbidity burden, exhibited a shorter hospital stay. This is clinically noteworthy as patients with higher Charlson and Elixhauser scores are typically expected to have slower recovery and greater perioperative resource utilization. One possible explanation is that improved early construct behavior and reduced microinstability may facilitate mobilization and rehabilitation, thereby shortening hospitalization. However, this interpretation remains speculative. Length of stay is influenced by multiple factors, including discharge protocols, pain management pathways, rehabilitation availability, surgeon preference, and institutional logistics. Therefore, while this finding is clinically interesting, it should not be interpreted as definitive evidence of the causal superiority of the OI strategy [5,8,15].

Radiological findings provide additional context for the clinical outcomes. In this study, SVA was significantly lower in the OI-focused group at 6 and 9 months, suggesting better preservation of global sagittal alignment during follow-up. This observation may be clinically relevant, as sagittal alignment is strongly associated with energy expenditure, compensatory mechanisms, disability, and health-related quality of life in degenerative and reconstructive spine surgery [11,12,13,14,17,18]. Contemporary reviews emphasize that sagittal balance is not merely a radiographic parameter; even mild postoperative malalignment can contribute to persistent symptoms and mechanical overload, particularly in multilevel lumbar procedures [12,13,14,17]. Thus, the lower SVA values observed in the OI-focused cohort may represent a biomechanically advantageous pattern consistent with the improved pain and disability scores seen during the same period.

The interpretation of LL and SS requires greater caution. Although statistically significant differences were observed at later time points, these parameters should not be interpreted in isolation or within a simplistic “higher is better” framework. Modern alignment concepts emphasize patient-specific targets, including age-adjusted alignment goals and the relationship between pelvic morphology and lumbar curvature [12,13,14]. In degenerative lumbar surgery, both the magnitude and distribution of lordosis are important. Therefore, the observed differences in LL and SS at 9 months do not necessarily indicate absolute radiographic superiority of one construct over another. Rather, they suggest that OI-focused stabilization may influence postoperative alignment patterns, a finding that warrants further investigation using more comprehensive spinopelvic models and longer-term follow-up.

Fusion-related outcomes should be interpreted with particular caution. In the revised analysis, Bridwell grades are presented in a standard table with exact numbers, percentages, and exploratory Mann–Whitney U *p*-values. However, the assessment was based on plain radiographs, and CT-based fusion analysis, which would provide a more robust evaluation in the presence of metallic instrumentation, was not uniformly available. Although 9-month plain-radiographic Bridwell grades were available, these results remain exploratory because CT-based confirmation and longer-term fusion assessment were not available. Therefore, the fusion-related findings are presented as supportive and exploratory rather than as definitive evidence of fusion superiority.

The present findings are consistent with a growing body of literature suggesting that biologically active or osteoconductive implant strategies may improve the quality of the bone–implant interface in spinal surgery. Experimental and clinical studies have demonstrated that bone graft-augmented titanium architectures may enhance osseous integration by increasing surface area, facilitating cellular attachment, and enabling bone ingrowth within implant scaffolds [5,6,7,8,15,16]. Although this study evaluated posterior stabilization rather than isolated screw design, similar biological principles may plausibly have contributed to the more favorable mid-term outcomes observed in the OI-focused group. Because several cited studies primarily evaluate interbody cages or general titanium biomaterial behavior rather than pedicle screw osseointegration specifically, these references are used to support general osteoconductive and bone-ingrowth principles; extrapolation to screw fixation is indirect and should be interpreted cautiously. Nevertheless, the retrospective design, non-randomized implant selection, limited sample size, and short follow-up period preclude any conclusion that the two-stage allograft-augmented strategy is universally superior to conventional PSS. Therefore, the current findings should be considered promising but preliminary.

Several methodological limitations should be emphasized. First, the study design does not allow attribution of the observed benefits to a single component of the OI-focused strategy. The term “OI-focused stabilization” reflects a broader treatment protocol involving allograft augmentation, staged rod placement, implant characteristics, and intraoperative decision-making, rather than directly verified osseointegration. Second, complete BMD/DXA data were not available for all patients, limiting objective evaluation of osteoporosis severity despite the clinical relevance of bone quality for screw loosening and fusion. Third, formal inter-observer reliability statistics, including ICC, were not calculated because separate observer-level datasets were not uniformly preserved. Fourth, the sample was imbalanced (PSS: *n* = 28; OI-focused: *n* = 12), which reduces statistical power, increases the risk of type I and type II errors, and limits the generalizability of the conclusions. Fifth, multiple exploratory comparisons were performed without formal adjustment for multiplicity; therefore, statistically significant findings should be interpreted cautiously. Sixth, multivariable adjustment for potential confounders, including comorbidity burden, was not performed because the available sample size would not support stable regression models. Seventh, the available retrospective aggregated dataset did not allow uniform reconstruction of standard deviations and effect-estimate 95% confidence intervals for all variables; importantly, confidence intervals were not assigned to *p*-values because *p*-values themselves do not have confidence intervals. Finally, the 9-month follow-up period is relatively short for evaluating mature fusion, recurrent loosening, hardware failure, and revision surgery rates. The G*Power (Version 3.1.9.7)-compatible sensitivity analysis indicated that the present cohort was powered only to detect large between-group effects, reinforcing that the findings should be interpreted as exploratory and hypothesis-generating.

Despite these limitations, the study provides clinically meaningful comparative data in an area where direct evidence remains limited. Much of the contemporary literature on OI in spine surgery focuses on biomaterials, interbody cages, and preclinical implant science rather than patient-level comparative outcomes in multilevel degenerative surgery [5,6,7,8,15,16,19]. In this context, the present findings are valuable, as they suggest that OI-focused constructs may translate into measurable improvements in pain, disability, and selected sagittal alignment parameters at mid-term follow-up. These data support further investigation and may contribute to hypothesis generation for future controlled studies.

Future research should prioritize adequately powered prospective comparative designs incorporating standardized definitions of allograft augmentation and OI-focused fixation, longer radiographic follow-up, patient-level CT-based fusion assessment, and formal inter-observer reliability analysis. The inclusion of BMD/DXA measurements, evaluation of screw loosening and subsidence, age-adjusted sagittal alignment targets, and subgroup analyses based on osteoporosis status, smoking, diabetes, and construct length would strengthen mechanistic interpretation and help identify patients most likely to benefit from this strategy [9,10,11,12,13,14].

## 5. Conclusions

A two-stage allograft-augmented fixation strategy intended to promote biological fixation was associated with improved mid-term pain control, better functional recovery, and more favorable sagittal vertical axis values compared with conventional PSS in this retrospective cohort of patients undergoing multilevel surgery for degenerative spinal pathology. However, these findings should be interpreted with caution due to the limited and imbalanced sample size, retrospective nature of the non-randomized design, selection bias, multiple exploratory comparisons, absence of direct osseointegration measurement, absence of multivariable adjustment, incomplete BMD/DXA characterization, lack of uniform CT-based fusion analysis, absence of formal ICC analysis, short follow-up duration, and limited ability to reconstruct standard deviations and effect-estimate confidence intervals for all variables from the retrospective aggregated dataset. These results do not establish causal superiority of the two-stage allograft-augmented strategy and should not be interpreted as definitive evidence of superior osseointegration or fusion. Prospective controlled studies with adequately powered sample sizes, standardized CT-based fusion assessment, and longer follow-up are warranted.

## Figures and Tables

**Table 1:** Patient demographics.

Variable	PSS (*n* = 28)	OI-Focused (*n* = 12)	*p*-Value
Age, years	60.8	62.3	0.216
Sex			0.085
Male	12 (42.8%)	4 (33.3%)	
Female	16 (57.2%)	8 (66.7%)	
Surgical levels			0.071
4	6 (21.4%)	1 (8.3%)	
5	8 (28.6%)	5 (41.7%)	
6	7 (25.0%)	3 (25.0%)	
7	5 (17.9%)	2 (16.7%)	
8	2 (7.1%)	1 (8.3%)	
CCI	0.68	0.79	0.043 *
BMI, kg/m^2^	29.6	30.2	0.374
Smoking status			0.258
No	16 (57.1%)	7 (58.3%)	
Current	8 (28.6%)	4 (33.3%)	
Former	4 (14.3%)	1 (8.3%)	
ECI	1.32	1.81	0.028 *
In-hospital stay, days	5.7	3.1	0.035 *
Complications	4 (14.3%)	1 (8.3%)	0.972

BMI, body mass index; CCI, Charlson Comorbidity Index; ECI, Elixhauser Comorbidity Index; PSS, posterior segmental stabilization; OI-focused, two-stage allograft-augmented fixation strategy intended to promote biological fixation. Continuous variables are presented as mean values and categorical variables as *n* (%). *p*-values do not have confidence intervals; 95% confidence intervals apply to effect estimates where appropriate and calculable. * indicates statistical significance with *p*-value < 0.05.

**Table 2:** Functional outcomes.

Outcome	Time Point	PSS (*n* = 28)	OI-Focused (*n* = 12)	*p*-Value
VAS	Preoperative	8.12	8.22	0.564
VAS	1 month	3.45	3.91	0.132
VAS	6 months	2.21	1.47	0.026 *
VAS	9 months	2.19	1.46	0.023 *
ODI	Preoperative	54.48	56.72	0.316
ODI	1 month	26.53	28.32	0.361
ODI	6 months	21.68	17.12	0.038 *
ODI	9 months	21.54	17.11	0.043 *

VAS, Visual Analog Scale; ODI, Oswestry Disability Index; PSS, posterior segmental stabilization; OI-focused, two-stage allograft-augmented fixation strategy intended to promote biological fixation. Values are presented as mean values. *p*-values do not have confidence intervals; 95% confidence intervals apply to effect estimates where appropriate and calculable. * indicates statistical significance with *p*-value < 0.05.

**Table 3:** Sagittal parameters.

Parameter	Time Point	PSS (*n* = 28)	OI-Focused (*n* = 12)	*p*-Value
LL (°)	Preoperative	68.9	69.2	0.817
LL (°)	1 month	64.3	65.3	0.342
LL (°)	6 months	65.6	61.9	0.057
LL (°)	9 months	67.7	61.7	0.024 *
SVA (mm)	Preoperative	27.5	28.3	0.852
SVA (mm)	1 month	23.5	24.6	0.421
SVA (mm)	6 months	25.6	22.7	0.043 *
SVA (mm)	9 months	25.9	22.8	0.038 *
SS (°)	Preoperative	51.7	52.2	0.263
SS (°)	1 month	49.1	50.3	0.357
SS (°)	6 months	50.5	48.4	0.053
SS (°)	9 months	51.7	48.2	0.042 *
PI (°)	Preoperative	49.7	49.2	0.362
PI (°)	1 month	52.4	52.3	0.472
PI (°)	6 months	52.5	52.4	0.534
PI (°)	9 months	52.9	52.2	0.625

LL, lumbar lordosis; SVA, sagittal vertical axis; SS, sacral slope; PI, pelvic incidence. Values are presented as mean values. *p*-values do not have confidence intervals; 95% confidence intervals apply to effect estimates where appropriate and calculable. * indicates statistical significance with *p*-value < 0.05.

**Table 4:** Bridwell fusion grades on plain radiographs at 1-, 6-, and 9-month follow-up time points.

Time Point	Bridwell Grade	PSS, *n* (%)	OI-Focused, *n* (%)	*p*-Value
1 month	Grade I	18 (64.3%)	10 (83.3%)	0.205
	Grade II	7 (25.0%)	2 (16.7%)	
	Grade III	2 (7.1%)	0 (0.0%)	
	Grade IV	1 (3.6%)	0 (0.0%)	
6 months	Grade I	7 (25.0%)	8 (66.7%)	0.026 *
	Grade II	8 (28.6%)	2 (16.7%)	
	Grade III	9 (32.1%)	1 (8.3%)	
	Grade IV	4 (14.3%)	1 (8.3%)	
9 months	Grade I	5 (17.9%)	6 (50.0%)	0.043 *
	Grade II	6 (21.4%)	3 (25.0%)	
	Grade III	8 (28.6%)	1 (8.3%)	
	Grade IV	9 (32.1%)	2 (16.7%)	

Note: Bridwell table *p*-values do not have confidence intervals; 95% confidence intervals apply to effect estimates where appropriate and calculable. * indicates statistical significance with *p*-value < 0.05.

## Data Availability

The datasets generated and/or analyzed during the current study are not publicly available because they contain potentially identifiable patient information but are available from the corresponding author upon reasonable request.

## Abbreviations

OI	Osseointegration-focused two-stage allograft-augmented fixation strategy
BMI	Body Mass Index
CCI	Charlson Comorbidity Index
ECI	Elixhauser Comorbidity Index
LL	Lumbar Lordosis
ODI	Oswestry Disability Index
PI	Pelvic Incidence
PSS	Posterior Segmental Stabilization
SS	Sacral Slope
SVA	Sagittal Vertical Axis
VAS	Visual Analog Scale
